# Mercer's Hospital

**Published:** 1831-04-01

**Authors:** 


### MERCER'S HOSPITAL.

A CASE OF RUPTURED INTESTINE, WITH REMARKS ON SOME EFFECTS OF CONTUSIONS OF THE ABDOMEN. By JOHN HART, M. R. I. A., &ce.

[Dublin Hospital Reports, Vol. V.]

*Case.* July 12th, 1826. An hostler, aged 36 years, received a kick from a horse on the abdomen, at 11 o'clock, a. m., and was brought into Mercer's Hospital at six in the evening of the same day, complaining of excruciating pain over the whole abdomen, with constant vomiting. He was pale, anxious, pulse 140, lower extremities drawn up, abdomen flat and rigid, with extreme tenderness. He had been bled previous to admission, and some purgative medicine exhibited, which was thrown up by vomiting. He had made no water since the accident. A catheter drew off two pints. Leeches were applied to the abdomen, and an emollient enema exhibited. Loose stools were the consequence. The symptoms were not ameliorated, and the patient died at five o'clock on the following morning.

*Dissection.* "The peritoneum was suffused with numerous small red vessels, and coated over with a thin layer of fawn-coloured lymph, which could be easily scraped off, and in which no red vessels were visible; the intestines and omentum were agglutinated to each other, and to the inner surface of the abdominal parietes in several places, through the medium of this soft lymph; a quantity of serous fluid tinged with bile, and mixed with the contents of the intestines, was found within the sac of the peritoneum; this was altogether free from any fetid odour. On examining the intestines it was found that the effusion had taken place through a rupture in the upper portion of the jejunum, which divided this intestine half across; owing to the retraction of the coats of the intestine, this opening was of an oval form, and surrounded by a raised border consisting of the everted mucous membrane.* The lymph was in greatest abundance, and the adhesions were most extensive in the vicinity of this opening. There was a large dark-coloured patch on the arch of the colon, produced by an ecchymosis between its coats; but this intestine was unbroken although distended with fæces, some of which were nearly solid, as if they had been retained there for some length of time.

Between the layers of the transverse meso-colon and behind the root of the mesentery, there was a large quantity of dark coloured coagulated blood, amounting in quantity to about sixteen ounces. This blood had been shed from the smaller branches of the mesenteric vessels, as we ascertained by a careful examination that the aorta and all the large veins were uninjured."

Mr. Hart enters into a disquisition on rupture of intestine without breach of the abdominal parietes, because such an occurrence has not been satisfactorily accounted for hitherto. To our minds there is no great stretch of intellect required upon such an occasion. Whatever portion of intestine are not in a state of collapse, are filled more or less, with air or other matters liquid or solid. The parietes of the abdomen investing the intestines are elastic. Now we can conceive no more difficulty in the occurrence of a rupture of one of the intestines, under the operation of external violence, than the breaking of a bottle of wine in a hamper that had fallen off a cart, or was kicked by a horse's hoof. We are not convinced that "this particular kind of injury almost invariably happens to the jejunum, where it is continuous with the duodenum," because our memory serves us with more than one instance when blows on the abdomen ruptured other portions of intestine. We do not therefore attach very much importance to the following explanation given by Mr. Hart.

"Some other cause than simple distention must therefore operate in predisposing a certain portion of intestine to suffer the injury in question. It occurs to me, that this may more justly be attributed to the circumstance of the intestine being to a certain degree fixed on some resisting surface, at the mo-

* "As described by Mr. Travers in his work on Injuries of the Intestines."

ment when it receives the impulse of the contusing force. Such is the condition of the upper portion of the jejunum, which at its commencement is fixed to the spine, and which for some inches lower down is confined to the vicinity of that part by the shortness of its mesentery. The absence of such contiguous resisting surface, would explain why it was that the arch of the colon in the case here cited escaped being torn, although the violence had acted on it with a degree of force sufficient to rupture the blood-vessels between its coats, so as to produce extensive ecchymosis. Every part of the large intestine, however, is not so exempt from rupture produced from external violence, as are the arch of the colon and the more moveable part of the small intestines; the cæcum, for instance, which is usually bound down by the peritoneum to the surface of the right iliac fossa, is occasionally burst by external violence. There is a case of this kind related by Mr. Speer in the fourth volume of these Reports."

Some cases of rupture of the liver and spleen are alluded to by Mr. Hart, and also rupture of the urinary bladder. But on these we need not dwell. The following observations are extracted on account of their practical tendency.

### CONTUSION OF THE ABDOMINAL PARIETES.

"A contusion of the parietes of the abdomen may sometimes produce a degree of suffering, which might lead the observer to suspect at first, that rupture of the intestines had occurred. The most remarkable case of this kind which I recollect to have witnessed, occurred in a private of the Enniskillen Dragoons named Connor, whose horse fell and rolled over him at a review in the Phœnix Park on the 30th of July, 1821. When brought to the King's Infirmary, immediately after the accident, he was suffering great pain over the epigastrium, so considerable that he could not endure the slightest pressure; his breathing was anxious and hurried, but his pulse was not above 80, and full; he had no vomiting. He was much relieved by fomenting and leeching the abdomen, the use of an emollient enema, and rest for a few days, after which he speedily recovered and returned to his duty.

The symptoms which follow a rupture of an intestine with extravasation of its contents, are of such a complexion as to render the diagnosis between this injury and those other effects of contusions of the abdomen which I have here noticed, a matter of great difficulty. Its occurrence is indicated by the following symptoms; sudden acute pain mostly accompanied by fainting; the pain suffering no remission, but on the contrary, increasing in severity; vomiting sets in and continues until the termination of the case; the abdomen is flat and rigid, and intolerant of pressure; the countenance becomes deadly pale, and anxious, and the features shrunk; the pulse is small, feeble, and increased in frequency to 120 and upwards. In every case of this injury which I have seen there was retention of urine.* The patient generally dies within twenty-four or thirty-six hours after the accident; and on dissection there is discovered a rupture of the upper portion of the small intestine, or of some portion of the large intestine where it is fixed; there is extravasation of the intestinal contents, and lymph is extensively effused on the surface of the peritoneum, which in many places forms adhesions between the contiguous surfaces of the intestines."

Our author, though he cannot propose any plan of treatment based on experience of its efficacy, suggests that purgation must be improper, by its exciting the peristaltic action of the intestines, and thus increasing the effusion of their contents. Instead of purgatives, he would advise opiates, while local inflammation is to be counteracted

---

* "It is a curious fact, that retention of urine should follow rupture of the intestine, in other mammalia as well as man. Thus, in a Kangaroo which I have just dissected, and which animal died in consequence of ruptured intestine from an accident, the bladder was in a state of extreme distention."

by leeches or other sanguineous depletion, aided by counter-irritation. By these means, he imagines that sufficient time might be gained for the formation of adhesions around the wound of the intestine, by which the extravasated fluids might be circumscribed, and at length find their way along the intestine.

"Since the above was written, I have been informed that views respecting the treatment of this disease, somewhat similar to those I have arrived at, have also occurred to Dr. Graves and Dr. Stokes, who have communicated the following particulars respecting a case successfully treated on these principles in the Meath Hospital. A middle aged man was admitted on the 27th of June, 1830, apparently in the last stage of peritoneal inflammation. The disease was of three days' standing, had supervened suddenly, in a few days after hypercatharsis induced by a large dose of glauber salts, and followed by long continued exposure to cold. It was attended by several of the usual symptoms of peritonitis from ulcerative perforation of the intestine. The belly was swollen, and so exquisitely tender, that the slightest pressure made the patient utter screams. The countenance was hippocratic, and the patient tormented with constant hiccup. Coldness of the extremities had commenced and the pulse was weak and slow. Before the hour of visit, leeches had been applied to the belly without relief, the patient was then ordered one grain of opium every hour.

The next day it was found that the symptoms were improved. *The patient had not experienced the slightest coma, headach, or delirium.* The same plan of treatment was persevered in, the daily dose of opium being gradually diminished until the 7th of July, when the convalescence having been completely established, the remedy was omitted. During this time diarrhœa set in for three or four days severely; this was treated by the application of a few leeches to the anus, and the use of anodyne enemata.

The patient took in all one hundred and five grains of opium (exclusive of that in the injections) without ever experiencing any of the usual effects of this substance when exhibited in large doses!"

Experienced practitioners are well aware that, in certain diseases, especially those accompanied by much pain, opium may be given to an amount that would destroy life, if given in health—or in other kinds of diseases. Thus in tetanus, we have given tincture of opium in three drachm doses every four or five hours, without producing any more effects on the brain or nervous system than so much water! In such cases, the stupifying effects of opium appear to be *expended on the pained and irritated parts*, without being extended to the other parts of the living machine.

---

